# Ten hour donor heart ischemic time with 10ºC static storage

**DOI:** 10.1016/j.jhlto.2024.100163

**Published:** 2024-10-13

**Authors:** William Tucker, Yatrik Patel, Mark Petrovic, Chris Schwartz, Brandon Petree, Steve Devries, Brian Lima, John Trahanas, Matthew Bacchetta, Ashish Shah, Swaroop Bommareddi

**Affiliations:** a Vanderbilt University Medical Center, Department of Cardiac Surgery, Nashville, Tennessee; b Vanderbilt University Medical Center, Office of Transplant Procurement and Preservation, Nashville, Tennessee

**Keywords:** heart transplant, prolonged ischemia, heart failure, organ preservation

## Abstract

Utilization of 10ºC static storage safely extended both ischemic time and travel radius in heart transplantation. A 57-year-old man with ischemic cardiomyopathy, a left ventricular assist device (LVAD), and end-stage renal disease was listed for combined heart-kidney transplant. The donor hospital in Anchorage, AK, was located approximately 2,700 nautical miles and 8 hours from the recipient center. The organ was transported in 10ºC static storage with over 10 hours of ischemic time and had superb early allograft function. Excellent outcomes with extended ischemic times can be achieved without machine perfusion, provided good recovery, storage, and implant techniques are followed.

Cardiovascular disease remains a major cause of morbidity and mortality worldwide. By the year 2030, it is estimated that 8 million people will be affected by heart failure despite advances in both medical and surgical therapies.^1^ Heart failure related mortality has returned to levels not seen since 1999 and while heart transplantation remains the gold standard curative therapy for patients with end-stage heart disease, the donor shortage persists.^2^, ^3^ Innovations in donor-recipient matching, extended criteria donor use, procurement and preservation techniques, and changes to the United Network for Organ Sharing (UNOS) organ allocation system have been implemented to alleviate the rising burden of end-stage heart failure and improve donor heart utilization.^4^ Historically, cardiac allograft ischemic times beyond 4–6 hours were deemed prohibitive due to increased mortality and higher rates of primary graft dysfunction (PGD).^5^, ^6^, ^7^ Donor heart ischemic times have restricted the recovery radius for transplant centers, limiting options for recipients. Efforts to overcome the limitation of prolonged ischemic times and travel distances have primarily been achieved with the use of expensive, single-use devices.^8^, ^9^, ^10^, ^11^, ^12^, ^13^, ^14^ Here we describe the use of a simple, multi-use 10ºC static storage system to facilitate the safe preservation of a donor heart for 10.35 hours of total ischemic time with excellent allograft function in a complex multi-organ LVAD explant recipient.

## Case presentation

**Figure 1:**
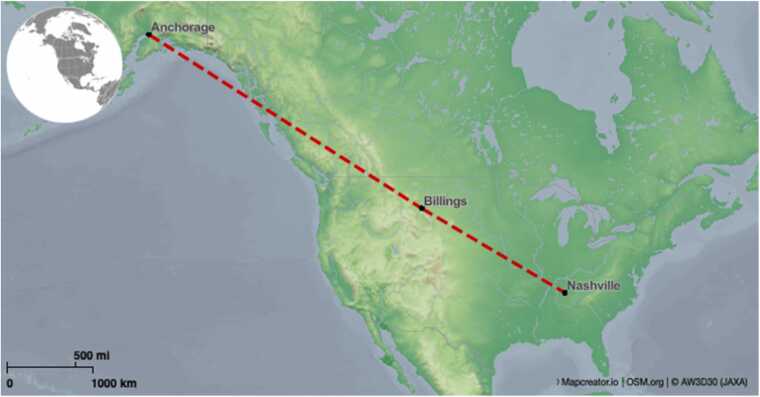
Flight map from Anchorage, AK to Nashville, TN.

**Figure 2:**
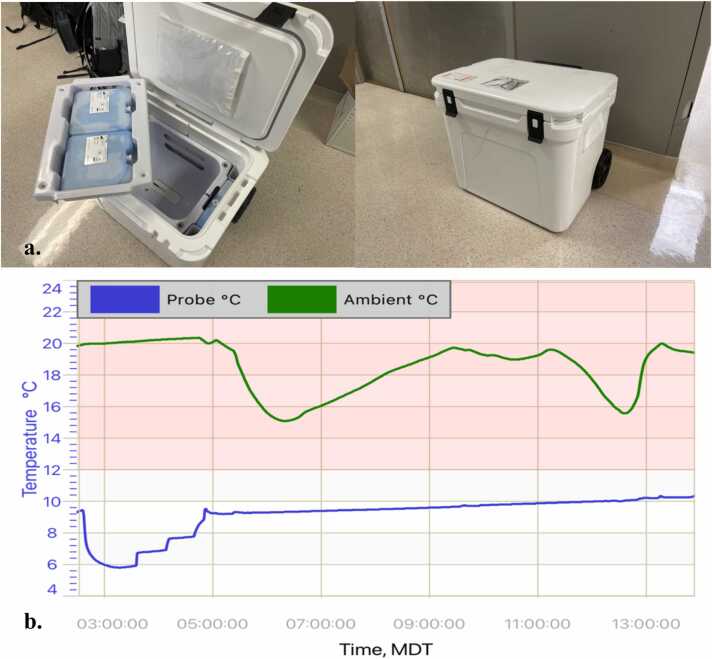
TenºC storage system with gel packs (a) and temperature log for donor heart in transit. Average temperature during cooler storage was 9.13ºC (b). Aortic cross-clamp applied at 0433 CST and reperfusion begun at 1454 CST.

A 57-year-old man with ischemic cardiomyopathy status post durable LVAD placement 1 year prior, end-stage renal disease on peritoneal dialysis, type 2 diabetes, and a history of left-sided stroke without residual deficits presented for transplant workup. After evaluation for heart-kidney transplant in the outpatient setting, he was listed for dual organ transplant. A suitable brain dead, 31-year-old donor was identified in Alaska, approximately 2,700 miles and over 8 hours from our hospital (Figure 1). Recovery was performed in our usual fashion using cold antegrade del Nido cardioplegia and the heart was placed into our 10ºC storage system (TRAFEROX – Toronto, CA) for transport and temperature monitoring (Figure 2).

The recipient underwent a challenging re-do sternotomy given the presence of adhesions from both his remnant temporary right ventricular assist device grafts and his LVAD. Cardiopulmonary bypass was initiated, followed by cardiectomy and LVAD explant. A donor patent foramen ovale was closed on the back table, followed by standard bicaval implantation. Empiric inotropic support was initiated and 30 minutes after reperfusion the patient separated from cardiopulmonary bypass uneventfully. The allograft total ischemic time was 621 minutes (10.35 hours), with 49 minutes of warm ischemic time. The patient did not require mechanical circulatory support post-operatively and demonstrated excellent allograft function.

**Figure 3:**
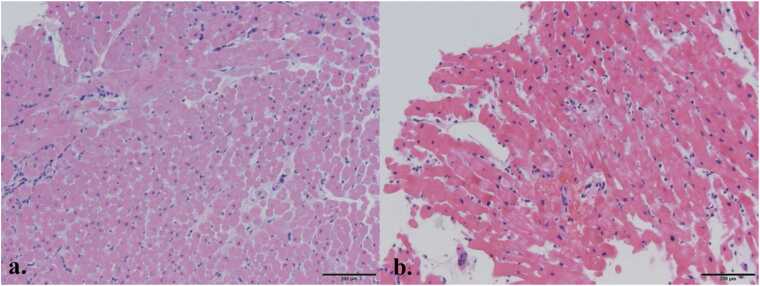
Endomyocardial biopsy H&E stain from POD 11 demonstrating mild-moderate interstitial edema and grade 1R acute cellular rejection (a). Second endomyocardial biopsy from POD 22 demonstrating no residual procurement effect and no evidence of rejection (b). POD, postoperative day.

Upon arrival to the cardiovascular intensive care unit, his cardiac index was 3.19 with a mixed venous saturation of 61%. His 24-hour vasoactive inotrope score was 22.3 and his 72-hour vasoactive inotrope score was 9.19. He underwent a kidney transplant on postoperative day (POD) 1 with good renal function subsequently. He transferred to out of the cardiovascular intensive care unit on POD9. His most recent echocardiogram demonstrated normal LV function with mildly reduced RV function. His endomyocardial biopsies demonstrated mild interstitial edema with mild acute cellular rejection on POD11 and no evidence of rejection on POD22, respectively (Figure 3). He was discharged from the hospital on POD24 without any incidence of severe PGD.

## Discussion

This is the first report of successful static preservation of a heart with a total ischemic time greater than 10 hours in a multi-organ transplant. Utilization of 10ºC static storage allowed for an optimal donor-recipient match and excellent allograft function without traditional distance and time limitations for a complex dual organ LVAD explant, heart-kidney transplant.

With a limited cardiac donor pool and increasing numbers of patients with end-stage heart failure, improvements and innovation in donor assessment, recovery, and organ preservation are necessary to increase the availability and quality of allografts. The current standard of care donor heart recovery involves hypothermic arrest with cardioplegia solution followed by storage and transport at a temperature of approximately 4ºC.^15^ This standard for organ recovery is based on principles of cold ischemic storage with the objectives of hypothermic metabolic arrest, preservation of the physical and biochemical environment for cellular integrity, and minimization of ischemia-reperfusion (IR) injury.^16^ Unfortunately, cold ischemic storage at 4ºC only allows for 4–6 hours before reperfusion is associated with increasing rates of PGD and heightened mortality. For this reason, anticipated travel times of over 4–6 hours often preclude donor consideration despite what would be an otherwise appropriate match. In attempts to extend ischemic times and expand the donor pool some variety of tightly controlled static cold storage, normothermic ex-vivo machine perfusion, or more recently hypothermic oxygenated perfusion has been used.^9^, ^12^, ^17^ Certainly, the use of the SherpaPak system, with its goal temperature of 4–8ºC for organ storage and improvements in rates of post-transplant mechanical circulatory support and severe PGD, has provided a stepping stone to investigate warmer organ storage temperatures clinically.^18^

Basic science research dating from over 30 years showed the optimal heart storage temperature may be 8–12ºC, which balances preservation of energy substrate, enzymatic function, and cellular integrity.^19^, ^20^ Thoracic transplantation has shifted focus from hypothermic metabolic arrest to bioenergetic homeostasis in organ preservation as our understanding of IR injury grows. Mediated in part by an imbalance in reactive oxygen species (ROS) and mitochondrial permeability transition pore (MPTP) opening, it appears that efforts to maintain mitochondrial integrity during preservation can mitigate the severity of IR injury and some of its downstream effects.^21^, ^22^ A growing body of clinical and basic science research in the field of lung transplantation has convincingly demonstrated improvements in allograft function and mitochondrial health with storage at 10ºC versus conventional storage at 4ºC.^23^, ^24^

Based on this information and our as of yet unpublished laboratory work, our group began utilizing 10ºC static storage for heart allografts almost exclusively in July 2023 with good outcomes, and we have previously reported a successful heart-lung transplant following prolonged ischemic time of almost 10 hours utilizing 10ºC static storage.^25^, ^26^

This case demonstrates the successful use of 10ºC static storage cardiac preservation with both a prolonged ischemic time and over 2,700 nautical miles traveled. It suggests that excellent outcomes with extended ischemic times can be achieved without machine perfusion, provided good organ recovery, storage, and implant techniques are followed. Extended preservation at 10ºC may prove to be a disruptive force in thoracic transplantation, alleviating geographic limitations and potentially allowing for improved and more expeditious donor-recipient matching. While further investigation is needed to better understand optimal donor heart preservation, with a particular focus on mitochondrial integrity, this case represents a step toward sustainable expansion of the donor heart pool.

## Glossary

LVAD: left ventricular assist device,
UNOS: united network for organ sharing,
PGD: primary graft dysfunction,
RVAD: right ventricular assist device,
MCS: mechanical circulatory support,
CVICU: cardiovascular intensive care unit,
VIS: vasoactive inotrope score,
POD: postoperative day,
IR: ischemia reperfusion,
ROS: reactive oxygen species,
MPTP: mitochondrial permeability transition pore
